# The Use of Machine Learning Methodologies to Analyse Antibiotic and Biocide Susceptibility in *Staphylococcus aureus*

**DOI:** 10.1371/journal.pone.0055582

**Published:** 2013-02-19

**Authors:** Joana Rosado Coelho, João André Carriço, Daniel Knight, Jose-Luis Martínez, Ian Morrissey, Marco Rinaldo Oggioni, Ana Teresa Freitas

**Affiliations:** 1 INESC-ID/IST, Technical University of Lisbon, Lisbon, Portugal; 2 Instituto de Microbiologia/Instituto de Medicina Molecular, Faculdade de Medicina, Universidade de Lisboa, Lisbon, Portugal; 3 Quotient Bioresearch, Fordham, United Kingdom; 4 University of Western Australia, Queen Elizabeth II Medical Centre, Nedlands, Australia; 5 Departamento de Biotecnologia Microbiana, Centro Nacional de Biotecnologia-CSIC, Madrid, Spain; 6 IHMA Europe Sàrl, Epalinges, Switzerland; 7 Dipartimento Biotecnologie, Università di Siena, Siena, Italy; University of Calgary, Canada

## Abstract

**Background:**

The rise of antibiotic resistance in pathogenic bacteria is a significant problem for the treatment of infectious diseases. Resistance is usually selected by the antibiotic itself; however, biocides might also co-select for resistance to antibiotics. Although resistance to biocides is poorly defined, different *in vitro* studies have shown that mutants presenting low susceptibility to biocides also have reduced susceptibility to antibiotics. However, studies with natural bacterial isolates are more limited and there are no clear conclusions as to whether the use of biocides results in the development of multidrug resistant bacteria.

**Methods:**

The main goal is to perform an unbiased blind-based evaluation of the relationship between antibiotic and biocide reduced susceptibility in natural isolates of *Staphylococcus aureus*. One of the largest data sets ever studied comprising 1632 human clinical isolates of *S. aureus* originated worldwide was analysed. The phenotypic characterization of 13 antibiotics and 4 biocides was performed for all the strains. Complex links between reduced susceptibility to biocides and antibiotics are difficult to elucidate using the standard statistical approaches in phenotypic data. Therefore, machine learning techniques were applied to explore the data.

**Results:**

In this pioneer study, we demonstrated that reduced susceptibility to two common biocides, chlorhexidine and benzalkonium chloride, which belong to different structural families, is associated to multidrug resistance. We have consistently found that a minimum inhibitory concentration greater than 2 mg/L for both biocides is related to antibiotic non-susceptibility in *S. aureus*.

**Conclusions:**

Two important results emerged from our work, one methodological and one other with relevance in the field of antibiotic resistance. We could not conclude on whether the use of antibiotics selects for biocide resistance or *vice versa*. However, the observation of association between multiple resistance and two biocides commonly used may be of concern for the treatment of infectious diseases in the future.

## Introduction

Antibiotics are among the most successful therapeutic agents so far developed by humankind. They have been used for decades for the treatment of bacterial infections in humans and animals. However, the widespread use of these compounds has led to the selection of resistant microorganisms, and consequently, to a loss of efficacy for this family of drugs [1], [2]. This problem is further increased by the circulation of microorganisms among humans, animals and agricultural hosts creating the opportunity for the exchange of resistance genes/mechanisms directing the spread of resistance [3]. This is a public health problem on a global scale since antimicrobial resistance can result in increased disease burden, morbidity and mortality [4], [5]. The problem is compounded by the fact that the pace of introduction of new chemotherapeutic drugs in the market and in clinical practice has slowed in the last few years [6]. The consequence of this slowdown is that many antibiotics lose their treatment effect against organisms that have developed antibiotic resistance and the use of other drugs, which are often reserved for last line treatment, is required. In some cases, therapeutic options are limited to just one antibiotic.

Besides antibiotics, another group of antimicrobials that might be relevant in selecting for antibiotic resistant bacteria are biocides. Biocides, used in antiseptics, disinfectants and preservatives, have been widely employed to control the growth of microorganisms such as bacteria, fungi, protozoa or viruses [7]. Their use has increased in a wide range of applications, namely in the domiciliary and in the healthcare environments, food industry and industrial settings, and in veterinary environments [8].

In recent years, there has been considerable discussion and controversy on whether biocide and antibiotic decreased susceptibility are connected [6], [9], [10]. This was highlighted some years ago, with the remarkable discovery of a shared bacterial target (FabI) between a biocide (triclosan) and an antibiotic (isoniazid) [11]. In the same study, it was described that triclosan exposure was followed by mutations in this bacterial target, which led to antibiotic resistance. Furthermore, even for those biocides which do not share a target with any antibiotic, reduced susceptibility can be achieved either when two elements, one conferring antibiotic resistance and another conferring reduced susceptibility to biocides are present in the same mobile element (co-resistance), or when a single resistance determinant, such as a multidrug (MDR) efflux pump, can provide resistance to both antibiotics and biocides (cross-resistance).

In this regard, some authors have suggested that there is an association between biocide-resistance and enrichment of antibiotic-resistance genes [12]–[14] and others have described increased bacterial resistance to biocides in areas where they have been used [15]–[17].

Despite the fact that the term *resistance to biocides* has been used in some papers [18], [19], resistance to these compounds is still to be clearly defined, unlike the case of antibiotics. Chapman suggests that the definition of resistance to biocides should proceed in a manner analogous to the definition for antibiotic resistance [18]. For antibiotics, there are two definitions of resistance, either based on the clinical breakpoints or on the epidemiological cut-off values (ECOFFs) of the minimal inhibitory concentration (MIC) whose values may differ. The setting of clinical breakpoints is a widely debated topic among the scientific, regulatory, and industrial community. Clinical breakpoints are discriminatory antimicrobial concentrations used to classify isolates as resistant, intermediate or susceptible, on the basis of the relevance of these values for treatment of infections [20]. Breakpoints are defined based on many factors, namely pharmacokinetic, dose-effect relationships from *in* *vitro* studies and animal studies, available formulations, clinical indications and practices, and target organisms [21]. Moreover, Monte Carlo simulations have been applied to help in establishing clinical breakpoints [22]. On the other hand, ECOFFs are used to define the intrinsic susceptibility to antibiotics of a given microbial species based on the MICs of wild type populations [23]. The ECOFF is independent of clinical treatment and will not be altered by changing circumstances [21]. Under this definition, any isolate presenting a MIC above the ECOFF value of the agent in question is considered as being non-susceptible. In the case of biocides, to date, none of these definitions have been established and we still do not have any regulatory or industrial organization involved in the normalization of classical breakpoints or of ECOFF values, which is important for studying biocide reduced susceptibility and changes therein.

There are many studies on the evaluation and evolution of antibiotic resistance [24]–[27] and some on the efficacy of biocides [27]–[29]. However, studies comparing biocide and antibiotic resistance are less common and the data sets used in these studies are typically small. Some studies follow the approach of simple statistical analysis using bivariate correlations and tests for independence [30]–[33]. Another study [34] uses a simple statistical analysis approach and hierarchical clustering on a small data set to identify statistical correlations between bacterial patterns of reduced susceptibility for disinfectants and antibiotics. Another paper [35] describes the use of classical association tests as ANOVA, Spearman-Rho correlations and principal component analysis to investigate the existence of correlations between the biocides and the antibiotics tested. The authors do not conclude whether there is an association or not, but with the data and tools used in the analysis it is hard to conclude if increased biocide resistance is a cause of increased antibiotic resistance. All of these studies deal with small data sets and the results present no evidence of statistical correlation between those antimicrobials, either antibiotics or biocides.

In this paper, we propose a new framework to analyse and cope with the scale and complexity of biocide susceptibility data, antibiotic susceptibility data and patient demographic data. In one of the largest phenotype studies ever performed on biocide and antibiotic reduced susceptibility, a total of 1632 world-wide clinical strains of *Staphylococcus aureus* (*S. aureus*) strains were analysed. *S. aureus* is a major human pathogen, a major cause of nosocomial infections, and also a significant cause of food borne infection. We combined different machine learning methodologies, namely decision trees and clustering, to explore the data in order to find biological and statistical significant results. Machine learning methods can be very powerful in finding unexpected patterns and hidden knowledge as well as establishing new rules from large data sets [36], [37]. Furthermore, using this approach we established a framework of analysis that may help determine values for the ECOFF of two biocides commonly used in our daily lives.

## Materials and Methods

### Strain Collection

A total of 1632 *S. aureus* strains were analysed in this study, taken from the collection of Quotient Bioresearch (Fordham, UK) [15]. The strains were collected between 2002 and 2003, from different geographical origins (including Europe, East Asia, Oceania and both North and South Americas) and represented both hospital and community acquired infections.

### Antibiotic and Biocide Susceptibility Testing

The study was conducted using the Clinical and Laboratory Standards Institute (CLSI) standardised broth MIC and MBC (minimum bactericidal concentration) methodology [38], [39]. Application of CLSI procedures to the study ensured that MIC data was generated using internationally recognized and standardised methods. Biocide and antibiotic susceptibility was determined by measuring both bacteriostatic activity (MIC) and bactericidal activity (MBC). The MIC was determined as the lowest concentration of compound required to completely inhibit the growth of an organism. The MBC was determined as the lowest concentration of a compound required to produce a 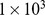 reduction in colony forming units [38], [39].

### Bivariate Correlations

A bivariate correlation analysis was performed between biocides and antibiotics’ variables. Spearman’s correlation coefficient was computed for each bivariate combination of these variables in order to find non-linear associations between the biological variables. Bivariate correlations were calculated using Matlab® 7.10.0.499 (R2010a). For each computed Spearman’s correlation coefficient, a hypothesis test was performed in order to test for statistical association between each pair of variables and a *p-value* calculated, assuming statistical significance at a level of 5%. When performing a set of multiple statistical inferences it is likely that a multiple testing problem of incorrectly rejecting the null hypothesis will occur [40]. To control the family-wise error rate, the classical conservative approach of Bonferroni correction was applied [41] leading to a critical value at level
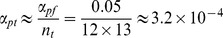
where 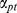 is the probability of Type I error (false positive) *per test*; 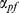 is the probability of Type I error *per family* of tests, also called, *familywise* or the *experimentwise* alpha; 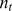 is the number of pairwise tests performed.

### Decision Trees with Permutation Tests validation and Visualisation

Within Machine Learning, Decision Tree algorithms are some of the most widely used for classification [37]. Learned trees have an intuitive form and represent a set of if-then rules. This method is generally very well accepted by domain experts since it is very simple to understand and interpret. For this particular case, decision trees were built using all the data as a training set. A decision tree inductor algorithm was applied in such a way that whenever it encountered a set of items (training set), it identified the attribute that discriminated the various instances most clearly. Features that lead to the highest information gain were successively chosen to build the decision tree. Trees complexity was controlled by a pruning stopping criteria. In the context of this analysis, the objective was to apply a pruning that could generate less complex decision trees: on the one hand it would have better readability, and on the other, it would include enough features to explain the data. Leaves were allowed to have a minimum number of objects of 1% of the data set dimension.

Thirteen different decision trees were obtained, one for each different antibiotic. The antibiotic classification (Susceptible or Non-Susceptible) was selected as the dependent variable whereas the independent variables included MIC and MBC values for the four biocides’, the year and region of collection, the age and gender of the host, the source and type of infection as well as the status of the host: hospital inpatient or outpatient. Decision trees were produced using Weka (Waikato Environment for Knowledge Analysis) Version 3.6.4.

rmine whether the observed decision trees is due to the randomness introduced in the sampling or not [42].These are non-parametric statistical tests for determining statistical significance by rearranging the labels of a data set.Permutation tests can help reducing the multiple testing burden [43] and can be used to compare statistical tests [44].

ngement of the labels is performed *n times* and the values of the statistic in use are calculated giving rise to a distribution of permutation values.On the other hand, a test statistic is computed from the experimental data set and then compared with the distribution of permutation values previously obtained. The main drawback of this method is that it needs a very large number of permutations to accurately estimate small p-values, a procedure that is computationally expensive [45].

A hypothesis test was performed where the null hypothesis, *H_0_*, represents the absence of the effect given by the decision tree profile obtained. Small p-values are evidence against the null hypothesis and in favour of a real effect in the population. The statistic used is based on the conditional probability of the Fisher’s Exact Test [46].

In order to visualise the distribution of the antibiotic resistance patterns found in the dataset and its relationships with the biocide MICs, graphic matroids were created. Graphic matroids are representations of unrooted trees similar to a minimum spanning tree but using a set of rules instead of distances. For each isolate, a pattern of susceptibility was obtained by binary coding the antibiotic susceptibility of each of the 13 antibiotics. Each node on the resulting tree represents a unique antibiotic susceptibility pattern found in the dataset. The graphic matroid was created using the goeBURST algorithm rules [47] with the different susceptibility patterns as an input. The size of each node is a function of the number of isolates found in the dataset with that pattern.PHYLOViZ software [48] was used to create the graphic matroid and the visualisation of the decision tree rules.

### Clustering

The goal of clustering is the separation of objects into groups or clusters using only the input vectors. This separation is based on a measure of similarity between objects [49], [50].

Hierarchical clustering was used to produce dendograms that could demonstrate co-resistance of antibiotics among the isolates. The measure of dissimilarity was based on the Euclidean distance metric and the hierarchical clustering method with average linkage was chosen. Clustering was obtained with Cluster 3.0 using the C Clustering Library version 1.50 and the visualisation was performed with Java TreeView 1.1.6r2.

## Results

**Figure 1:**
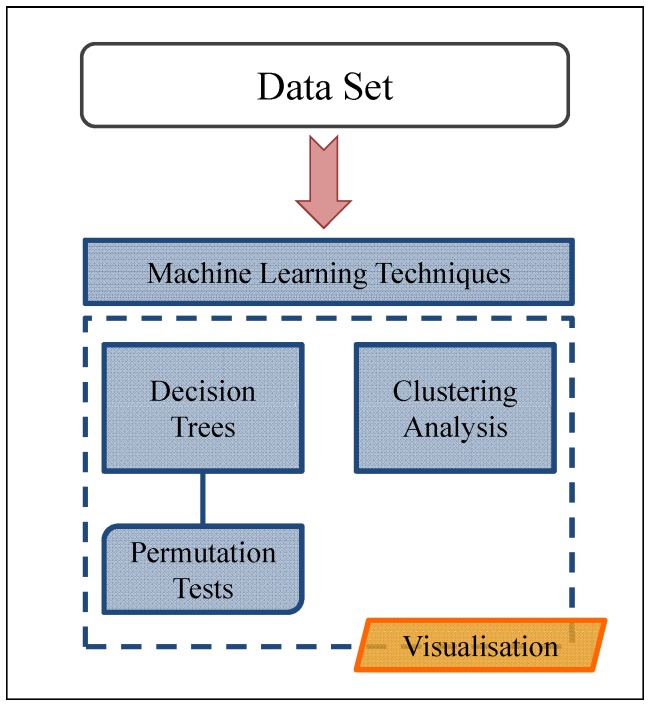
New framework of analysis. In this paper, we propose the use of machine learning techniques to analyse epidemiological data sets, namely decision trees validated by permutation tests and clustering analysis of the data set followed by a visualisation step of the results. This methodology is successful in dealing with the complexity of molecular epidemiology data and its application in studying the potential linkage between antibiotic resistance and biocide reduced susceptibility.

In this paper, we first performed the standard approach described in the literature [30]–[33] for epidemiological data analysis by calculating the bivariate correlations between biocide and antibiotic phenotypes. This approach failed to find any associations between the variables in analysis (data not shown). We then followed a different approach represented in Figure 1 by applying machine learning techniques, namely decision trees validated by permutation tests and clustering analysis of the data set followed by a visualisation step. We propose this new framework, based on machine learning methodologies, to analyse the scale and complexity of molecular epidemiology data and its application to study the potential linkage between antibiotic resistance and biocide reduced susceptibility.

### Generation of the Data Set

A collection of 1632 *S. aureus* strains isolated between 2002 and 2003, from different geographical origins, and representing both hospital and community acquired infections was analysed [15]. For all isolates, the MIC values of 13 different antibiotics, namely amoxicillin/clavulanate, cefuroxime, cefaclor, cefpodoxime, clindamycin, erythromycin, clarithromycin, azithromycin, telithromycin, ciprofloxacin, levofloxacin, gatifloxacin and moxifloxacin, were determined in addition to the susceptibility data for methicillin. A bacterial isolate was considered non-susceptible to an antibiotic agent when it tested resistant or intermediate when using clinical breakpoints as interpretative criteria according to the Clinical and Laboratory Standards Institute (CLSI) [51]. In addition, for all isolates, susceptibility data for four different biocides (MIC and MBC), namely chlorhexidine, benzalkonium chloride, sodium hypochlorite and triclosan, were obtained. Since there are no established breakpoints for biocides the information on the susceptibility to these compounds was introduced in the data set as actual MIC and MBC values and not as categories (susceptible or non-susceptible) like in the case of antibiotics. According to the classification of Magiorakos et al [52], strains were considered as multidrug resistant (MDR) when they were resistant to at least three different families of antibiotics. All the MIC and MBC values are in a log_2_ scale due to the methodology used to determine the phenotypic data.

A data set was generated which included, for each *S. aureus* isolate: the MIC value and the susceptible/non-susceptible classification for each of the 13 antibiotics; the MIC and MBC values for each biocide; the year (2002 or 2003) and region of collection (Europe, East Asia, Oceania and both North or South Americas); the age (ranging from 0 to 97 years old) and gender of the host; the source of the sample and infection type; and if the host was an inpatient or outpatient of the hospital were collection occurred.

### Bivariate Correlations

This is the standard approach reported in the literature to analyse data of epidemiological data sets. However, using this methodology there was no clear correlation between the biocide and antibiotic phenotype variables. Indeed, the data analysed showed weak bivariate correlations. This is a result that matches with previous studies of smaller data sets. After performing the correction for family-wise error rate, we found some pairs of variables (represented in bold in Figure S1, in supporting material) with a statistically significant Spearman’s correlation coefficient. In spite of having a statistically significant correlation coefficient, the pair of variables in bold have a considerably low absolute value for the coefficient so one cannot assume a strong correlation between those variables. The ratio between MBC and MIC (Fold Change) of each biocide was also analysed as this may indicate a difference in biocide reduced susceptibility. Kendall’s correlation coefficient was also computed for the same pairwise test variables and the results were similar (data not shown).

### Decision Trees

Since standard statistical tests did not enable us to obtain a clear picture of the relationships between antibiotic and biocide reduced susceptibilities, we decided to apply a different approach. The approach is shown in Figure 1 and is based on the application of machine learning methodologies that allow extraction of hidden knowledge and unexpected patterns from data as well as establishing new rules from large data sets [36].

**Figure 2:**
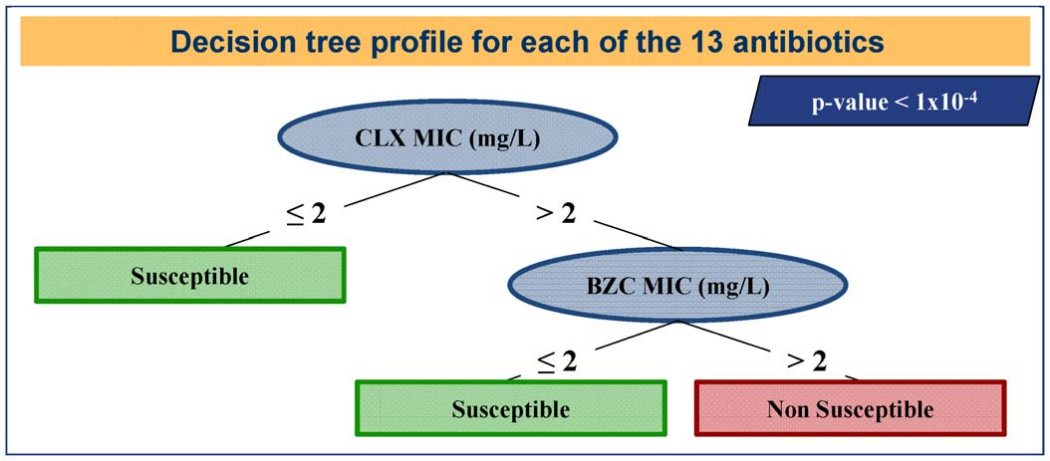
Decision tree profile for each of the 13 antibiotics. The level of the MIC of two common biocides is the best feature to predict the susceptibility and non susceptibility to each of the 13 antibiotics with a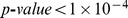.

After applying the tree induction algorithm to each of the 13 antibiotics under analysis, a common pattern emerged from the decision trees. The structure of these trees shows interesting relationships. Among all independent variables, the decision trees modelled the dependent variable based on two specific predictor variables as shown in Figure 2. Whereas most independent variables, including region and year of collection, were not the ones that better discriminate antibiotic susceptibility, the MICs of two common biocides chlorhexidine (CLX) and benzalkonium chloride (BZC) are the best predictors of susceptibility and non-susceptibility to each of the 13 antibiotics, with a 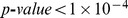, validated with permutation tests. This is a common profile to all 13 antibiotics and basically it can be summarized in two simple rules:

1. **MIC(CLX) ≤2** **mg/L:** strains are susceptible to the antibiotic in test
2. **MIC(CLX) >2** **mg/L 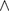 MIC(BZC) >2** **mg/L:** strains are non-susceptible to the antibiotic in test

The leaf **MIC(CLX) >2** **mg/L 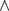 MIC(BZC) ≤2** **mg/L** involves a small number of strains, a total of 13 strains for each of the 13 antibiotics, representing less than 0.8% of the data set dimension.

The decision tree leaves have an average misclassification rate of 16% and 23% for the first and second rule, respectively. Addressing this misclassification rate, we have further analysed these isolates. In fact, the leaf of the first rule has an average of 84% true positives, which represent 74% of the total number of isolates of the data set. On the other hand, the second rule has an average of 77% correctly classified isolates representing 8% of the total number of isolates of the data set. Furthermore, the population of non-susceptible isolates is, on average, divided in a proportion of 2∶1 between the first and second rules.

In spite of not having strong correlations between any biocide or antibiotic variables, we have found interesting relationships between two specific biocides and all the 13 antibiotics analysed. This methodology allows finding out relationships other than non-linear monotonic ones. The CLX and BZC MICs that separate antibiotic susceptible from non-susceptible are very consistent among all the 13 antibiotics, 2 mg/L being the borderline for two biocides. These two biocides commonly used in our daily life belong to different structural families: Chlorhexidine a bisbiguanide and Benzalkonium Chloride a quaternary ammonium compound. Furthermore, using this approach we can suggest values for the ECOFF of Chlorhexidine and Benzalkonium Chloride, indicating a potential ECOFF of MIC <2 mg/L for the susceptible population and of MIC >2 mg/L for the non-susceptible strains for both biocides.

### Visualisation

**Figure 3:**
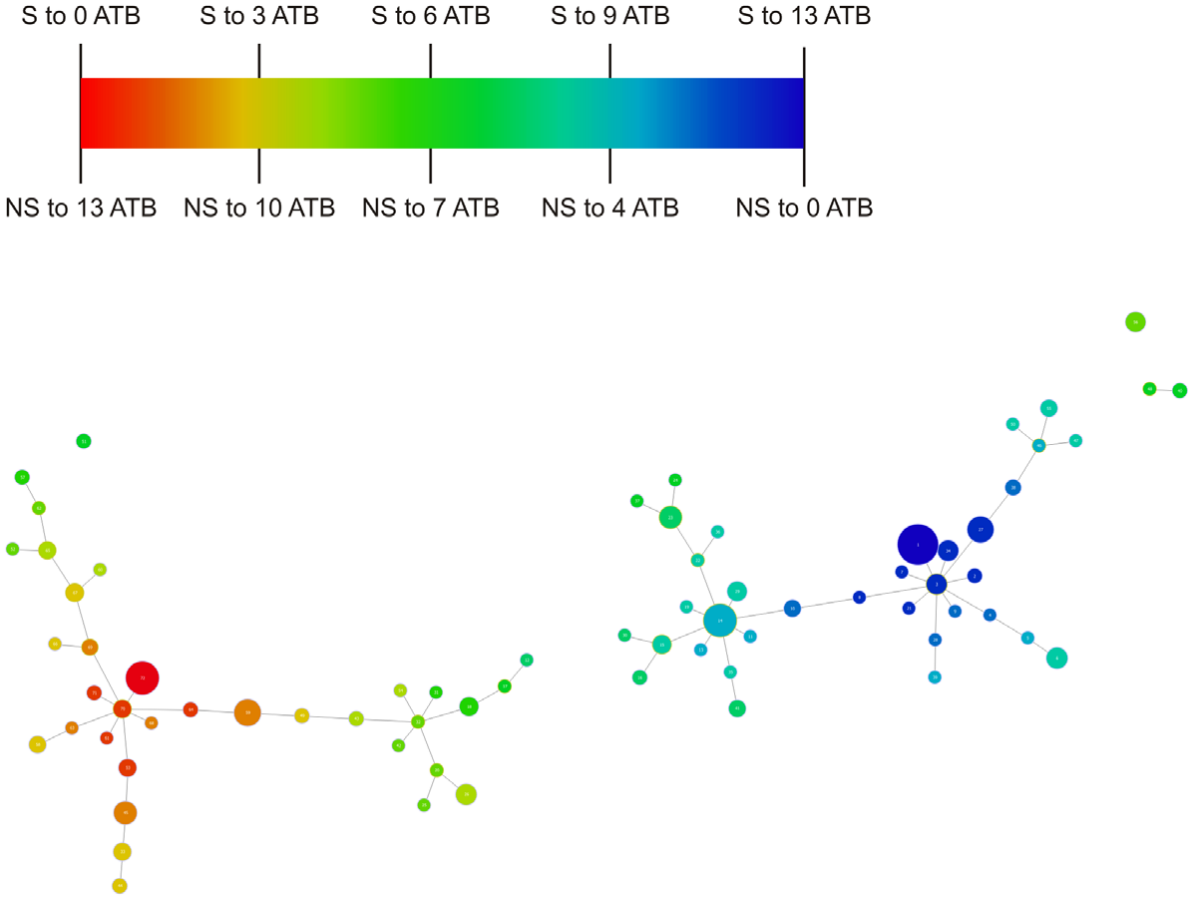
Graphic matroid of *S. aureus* antibiotic susceptibility. Graphic matroid of *S. aureus* antibiotic susceptibility with a red-blue colour map. Isolates in *blue* are susceptible to a greater number of antibiotics and isolates in *red* share a greater non susceptibility to antibiotics. S-Susceptible. NS-Non-susceptible. ATB-Antibiotics.

Figure 3 shows the distribution of the susceptibility patterns. Each link represents a unique change in the overall pattern. Individual clusters are produced that are different among themselves by two or more differences (variations) in the pattern. A Blue to Red colour map was created to help the visualisation of the overall antimicrobial resistance dispersal on the tree: the isolates in dark blue are susceptible to all of the antibiotics, while isolates in red share a greater non-susceptibility to antibiotics. The isolates in blue are clearly concentrated on the right-hand side of the graph and the isolates in red, on the left-hand side, represent a gradient of multidrug resistance in our dataset.

**Figure 4:**
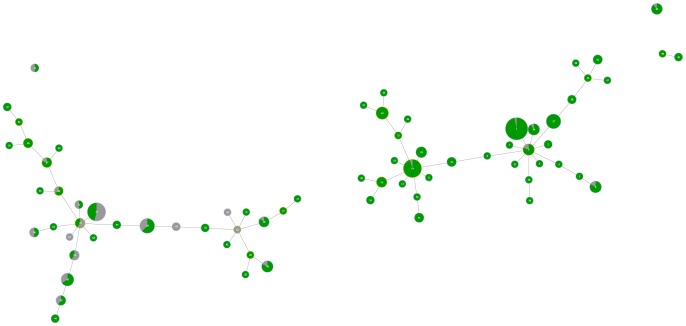
Graphic matroid of *S. aureus* isolates colour mapped based on the first decision tree rule. Isolates with CLX MIC≤2 (classified as susceptible to each antibiotic, based on the first rule) are coloured in *green*. In *grey* are represented the remaining isolates.

**Figure 5:**
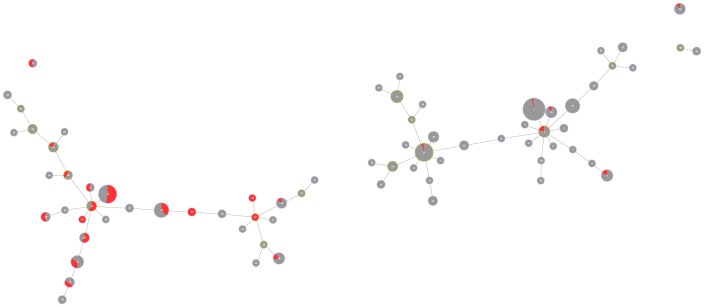
Graphic matroid of *S. aureus* isolates colour mapped based on the second decision tree rule. Isolates with CLX MIC >2
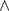
BZC MIC >2 (classified as non-susceptible to each antibiotic, based on the second rule) are coloured in *red*. In *grey* are represented the remaining isolates.

Based on this distribution of susceptible complexes we can also visualise each of the decision tree rules previously described. Since all the antibiotics have the same two rules obtained using the decision trees approach, we can colourmap each of these rules in order to visualise them in the graphic matroid. Figure 4 represents the isolates classified as susceptible to a given antibiotic in *green* based on rule **1**. They are predominantly present on the right-hand side of the graph, which corresponds to the region of antibiotic susceptible strains. There are some false positive strains represented in green on the left-hand side of the graph. Moreover, Figure 5 represents in red the isolates classified as non-susceptible based on rule **2**. These isolates are largely represented on the left-hand side of the graph; however, some isolates in red are also present on the right-hand side, corresponding to the susceptible antibiotic region of the tree.

### Clustering

We found two groups of antibiotic resistant strains: those that are misclassified as susceptible according with rule **1** and those that are correctly classified as non-susceptible according to rule **2**. To address whether these subpopulations represent specific subsets of non-susceptible strains they were further analysed. For this purpose, a hierarchical clustering analysis was performed on the two subpopulations of non-susceptible strains.

**Figure 6:**
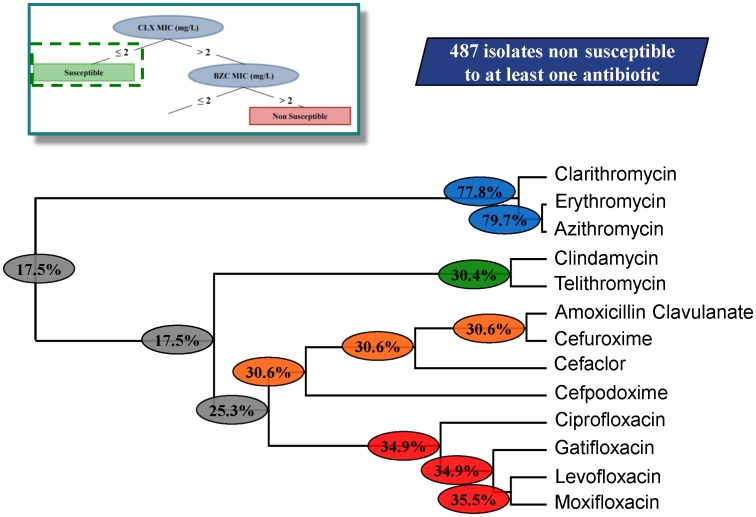
Clustering of the non-susceptible population for rule 1. The different clusters for the non-susceptible population of rule 1 are shown in the dendogram with the respective global percentage of shared resistance of each set of antibiotics. Among the 487 isolates eligible for the clustering analysis, only 17.5% isolates are non-susceptible to all the 13 antibiotics. We have identified 4 clusters of antibiotics which may be related with the potential mechanisms of resistance already reported on the literature [60].

**Figure 7:**
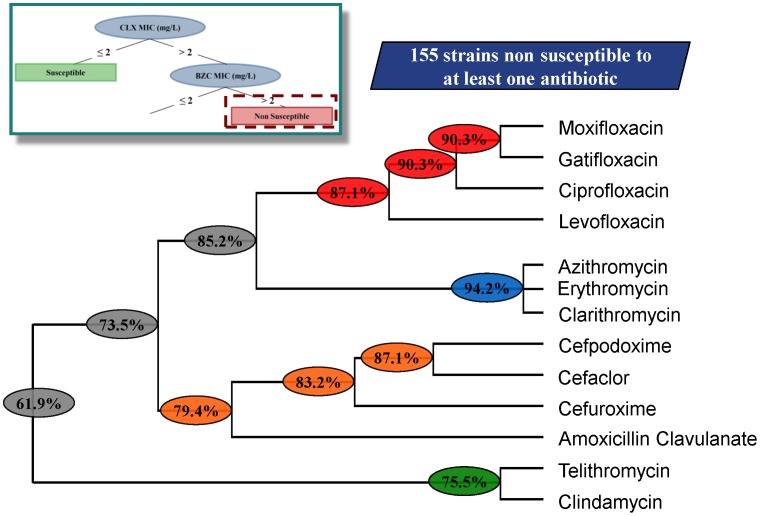
Clustering of the non-susceptible population for rule 2. The different clusters for the non-susceptible population of rule 2 are shown in the dendogram with the respective global percentage of shared resistance of each set of antibiotics. Among the 155 isolates eligible for the clustering analysis, 61.9% isolates are non-susceptible to all the 13 antibiotics. We have identified 4 clusters of antibiotics which may be related with the potential mechanisms of resistance already reported on the literature [60].

Of note here is that even for each single antibiotic whose number of misclassified strains is low according to rule **1** (in the range of 16%), the final percentage of isolates presenting resistance to at least one antibiotic is higher, about 34%. In agreement with this issue, the clustering of misclassified isolates of rule **1**, which are in fact non-susceptible isolates, shows that few of them have cumulative resistance to different antibiotic families (Figure 6) sharing only 17.5% of co-resistance. This result suggests that, for this group of non-susceptible strains, acquisition of resistance to one antibiotic is independent of the acquisition of resistance to another and is independent as well of the development of reduced susceptibility to the tested biocides. A different situation can be observed for the non-susceptible isolates, classified based on rule **2** (Figure 7). This population presents a high prevalence of strains, about 62%, displaying a phenotype of multiple resistance to the different families of antibiotics tested and presents as well reduced susceptibility to both biocides analysed.

## Discussion

The possibility that the use of biocides might be a risk for the selection of antibiotic resistant bacteria is a controversial issue still under discussion. Two fundamental concerns are raised: 1) there are some elements such as multidrug efflux pumps that can confer simultaneously resistance to antibiotics and biocides (cross-resistance) when they are over expressed, and 2) antibiotic resistance determinants transferred by horizontal gene transfer are usually associated to other determinants that are transferred together on the same genetic element, some of which are involved in resistance to biocides (co-resistance). In both situations, the presence of the biocide will select for antibiotic resistance and *vice versa.*

Several articles have shown the existence of the aforementioned linkage between antibiotic resistance and biocide reduced susceptibility [19], [53]–[59], however, most of these studies have been performed *in* *vitro* using model strains and the evidence of such association by means of epidemiological studies is much weaker. In fact, some papers that describe the use of classical association tests such as ANOVA, Spearman-Rho correlations and principal component analysis, arrive at the conclusion that “it is very difficult to support a hypothesis that increased biocide resistance is a cause of increased antibiotic resistance” [35]. The authors do not conclude whether there is or there is not an association, but that with the data and tools used in the analysis “it is very difficult” to reach such a conclusion.

In the case of the potential associations between biocides and antibiotic resistance, there are mainly two reasons for this lack of potential association. First, the number of isolates analysed in epidemiological studies with antibiotic and biocide susceptibility data is not usually high enough to get statistically robust conclusions. Secondly, performing these epidemiological studies highlights several difficulties due to the complexity of this type of data, mainly because of the large number of variables from different sources and the typical non-linearities of biological signals. To improve our current analysis, we have used decision trees validated with permutation tests for analysing biological data sets and in particular to unveil hidden associations between antibiotic resistance and biocide reduced susceptibility in *S. aureus*. These methodologies were successful in finding rules within the data set where the standard and most used statistical approaches found no associations. We studied the largest collection of isolates so far analysed for this purpose (1632 isolates). The main conclusion emerging from these unbiased analyses is that reduced susceptibility to two biocides, CLX and BZC, which belong to different structural families, is associated with resistance to several antibiotics, whereas resistance to just one antibiotic is not rare even in biocide-susceptible populations. A recent paper has proposed a classification of strains presenting resistance in three categories: multidrug resistant (MDR), when they present resistance to antibiotics belonging to three different families; extensively drug resistant (XDR), when they remain susceptible just to one or two families of antibiotics; and pan-drug resistant (PDR) when they are resistant to all known antibiotics [52]. Following these rules, 73.5% of isolates presenting low susceptibility to biocides are MDR *S. aureus* (Figure 7). The fact that neither the time nor the place of isolation were relevant predictors of resistance indicates that there is not a bias in the type of isolates used in our study.

As stated above, simultaneous reduced susceptibility to biocides can be achieved either by the acquisition of elements containing an array of genes, each one encoding resistance to a different drug, or by the overproduction of efflux pumps capable of extruding both biocides and antibiotics. Although we have not studied here the molecular basis of resistance of our isolates, our results strongly suggest that reduced susceptibility to CLX and BZC is associated to multidrug resistance in S. aureus. Whether this association is the consequence of biocide usage or it is a side effect of the selection of resistance by antibiotics remains to be established. It is relevant to note that using machine learning technologies we have been able to define two subpopulations with very different patterns of antibiotic non-susceptibility and the phenotype of two biocides, CLX and BZC, is enough to discriminate them. These populations were not apparent using more classical statistical tests. We have consistently found that a CLX MIC greater than 2 mg/L and a BZC MIC greater than 2 mg/L is related to antibiotic non-susceptibility in S. aureus. Given that those values distinguished between two populations presenting different phenotypes, it might be possible that these values represent cut-off values for biocide non-susceptibility. In this regard, our study may help establishing the ECOFF value of two biocides, CLX and BZC, commonly used in our daily lives. Nevertheless, the ECOFF values need to be confirmed with other types of analyses [15]. These studies are now in progress in our laboratories. Two important results, one methodological and another with relevance in the field of antimicrobial resistance emerge from our work. Machine learning methods are useful for the extraction of hidden knowledge and unexpected patterns from large datasets, such as those generated in our work, in situations in which more classical statistical tests may fail to find associations. Multidrug resistance is associated with reduced susceptibility to benzalkonium chloride and chlorhexidine in S. aureus, whereas this association is not observed at the level of resistance to a single antibiotic. From our results, we cannot conclude on whether the use of antibiotics selects for biocide resistance or vice versa. However, the observation of association between multiple resistance and two biocides commonly used may be of concern for the treatment of infectious diseases, in the future.

## Supporting Information

Figure S1
**Matrix with Spearman’s correlation coefficients between biocides phenotypes (MIC, MBC or fold change) and antibiotics MIC values.** *P-values* were also computed in order to find statistical significant associations. In *blue* and *bold*, we have the statistical significant associations, i.e., the correlation coefficients whose *p-value* is lower than the critical value 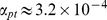.(TIF)
